# Inspiring the next generation: a qualitative study of rural healthcare professionals’ experiences as “books” in a living library

**DOI:** 10.3389/fmed.2026.1850674

**Published:** 2026-06-16

**Authors:** Grace Perez, Kaia Thauberger, Pariza Fazal, Eron Muel, Aaron Johnston

**Affiliations:** 1 Distributed Learning and Rural Initiatives, University of Calgary, Calgary, AB, Canada; 2 Cumming School of Medicine, University of Calgary, Calgary, AB, Canada; 3 Department of Psychology, University of Calgary, Calgary, AB, Canada; 4 Werklund School of Education, University of Calgary, Calgary, AB, Canada; 5 Faculty of Nursing, University of Calgary, Calgary, AB, Canada; 6 Department of Emergency Medicine, University of Calgary, Calgary, AB, Canada; 7 Department of Family Medicine, University of Calgary, Calgary, AB, Canada

**Keywords:** human library, narratives, premedical students, re-authoring, rural medical education, rural medicine, storytelling, undergraduate medical education

## Abstract

**Background:**

Rural medical education requires learning approaches that connect learners with the lived realities of rural practice. One structured approach is the Living Library model, using narratives of lived experiences of rural healthcare professionals as knowledge resource. The benefits on learner outcomes are well-established, such as increased empathy and improved understanding of social and professional contexts of health, but little is known on the impact of telling their stories on the storytellers. This study explored what it means for rural healthcare professionals to serve as “human books” in a Living Library.

**Methods:**

This qualitative study used a phenomenological approach to describe how human books experienced sharing their personal and professional stories to medical learners across all stages of training. Semi-structured interviews were conducted with human books and data were analyzed thematically to understand the meaning of this experience.

**Results:**

Three main themes emerged: (i) participants felt a strong sense of responsibility to share their experiences to inform and inspire future rural practitioners; (ii) story preparation was emotionally challenging and required psychological readiness; and (iii) storytelling offered personal benefits, including connection, reframing past experiences, and renewed sense of purpose and meaning in their work. Participants described being a “human book” as both educational and personally meaningful, affirming their professional identity as rural practitioners.

**Conclusion:**

The Living Library model is an innovative educational approach that offers dual benefits for learners and storytellers. Such narrative-based initiatives can enrich rural medical education by providing learners with authentic insights into healthcare in rural areas, while empowering the storytellers through the validation of their unique career paths. This can deepen rural healthcare professionals’ understanding of their roles within the community, fostering agency and resilience, thereby enhancing wellbeing and professional satisfaction. Living Libraries can complement traditional curriculum and support rural workforce development in Canada and other underserved settings.

## 1 Introduction

Life stories have long been used in medical education to support learning that goes beyond biomedical knowledge (1). Narratives help learners understand the human experience of illness and healthcare practice, particularly in complex and uncertain contexts. Listening to patient and clinician stories helps learners engage with the human dimensions of care, including emotion, values, meaning and uncertainty, across undergraduate and postgraduate training and can enhance empathy, reflective capacity, and understanding of professional values (2, 3). Physicians’ life stories offer learners insight into career pathways, professional challenges, and the realities of medical work beyond technical competence. Studies have demonstrated that physicians’ narratives in undergraduate medical education can support professional identity formation and help learners make sense of what it means to become a doctor (4). Narrative approaches are widely accepted across undergraduate, postgraduate, and continuing medical education, using methods such as reflective writing, patient storytelling, illness narratives, and digital stories (5–7).

One structured approach to storytelling is the Living Library. This approach originated in Denmark in the 2000s, to bring people together to challenge prejudice and promote dialogue (8, 9). The purpose is to replace stereotypes with human connection and understanding. In this model, people become “human books” who share their lived experiences with “readers” through facilitated conversations. Like a regular library, readers “borrow” books, but the reading is the personal interaction between human books and readers through narratives of experiences and personal stories (10). Living Libraries have since been adapted across educational and professional settings, including health professions education (11–15). In these settings, “human books” may include patients, clinicians, or community members who share experiences related to illness, marginalization, or professional life, and were largely focused on reader outcomes, showing that Living Libraries can deepen understanding of patient perspectives, social contexts of health, and diverse career pathways (13, 15–17).

Storytelling is especially relevant in rural medical education as many learners have limited exposure to rural practice (18). Narratives from rural clinicians have been used to provide insight into the realities of rural life and highlight the complexity, rewards, and meaning associated with rural healthcare work. Perez and colleagues have shown that narratives from rural practitioners can be a powerful tool to challenge learners’ assumptions about rural practice and influence interest in rural careers. By sharing personal stories, clinicians offer learners an authentic understanding of rural healthcare that cannot be conveyed through curriculum content alone (19). With the growing use of Living Libraries in health professions education, the benefits on learners are well-described (16, 19), but little is known about how storytelling affects the “human books” themselves. Human books are central to the Living Library model, yet research has tended to focus on what readers gain, rather than what it means to repeatedly share personal stories. Human books are often regarded as educational resources rather than active participants whose own emotions and reflections may be affected by storytelling. Emerging work suggests that being a human book can be meaningful and affirming, offering a sense of renewed purpose and connection (20). However, these experiences remain underexplored in medical education research, particularly for rural healthcare professionals.

In the Living Library setting, stories are shared in conversation, often multiple times, and in response to learners’ questions. This can create opportunities for connection, validation, and contribution, but may also involve emotional vulnerability. Rural clinicians often work in settings characterised by professional isolation, close community relationships, and strong ties between personal and professional identity. Storytelling in this context may have unique emotional and relational impacts. There is limited research regarding the impact of storytelling on storytellers themselves, especially rural healthcare professionals participating in Living Libraries within medical education. Understanding these experiences is essential to ensure that narrative-based educational practices are reciprocal, ethical, and sustainable. Narrative theory suggests that telling and retelling stories can shape identity, emotional processing, and meaning-making (21–23). Through processes often described as re-authoring or re-storying, individuals may reinterpret past experiences, reshape their understanding of self and experience over time, integrate challenges and construct renewed purpose (24, 25). Exploring how storytelling influences emotional reflection, meaning-making, and relational outcomes for human books can deepen understanding of storytelling not only as a teaching strategy, but also as a form of reflective and professional practice. Addressing this gap supports the thoughtful integration of Living Libraries into rural health education and recognises the value of stories for those who share them, as well as for those who listen.

The authors are from a medical school in Western Canada that facilitates rural educational experiences to encourage rural healthcare interest among medical students and have adapted the Living Library model to provide learners with a better understanding of rural life and practice (19, 26). This study aimed to explore how rural health professionals experienced telling their personal and professional stories as human books, particularly on the emotional, meaning-making, and relational impacts of storytelling.

## 2 Methods

### 2.1 Theoretical framework

This qualitative study was guided by a phenomenological research approach. Phenomenology focuses on lived experience and how individuals make sense of a phenomenon in their everyday lives, aiming to understand meaning rather than measure behaviors or outcomes (27, 28). Being a human book in a Living Library is understood not simply as a teaching role, but as a personal and relational experience shaped by storytelling, professional identity, and interaction with learners. A phenomenological approach allows close attention to how rural healthcare professionals perceive, feel, and reflect on the act of telling their stories, consistent with the aim of describing the meaning of an experience from the perspective of those who have lived it (29).

**Figure 1:**
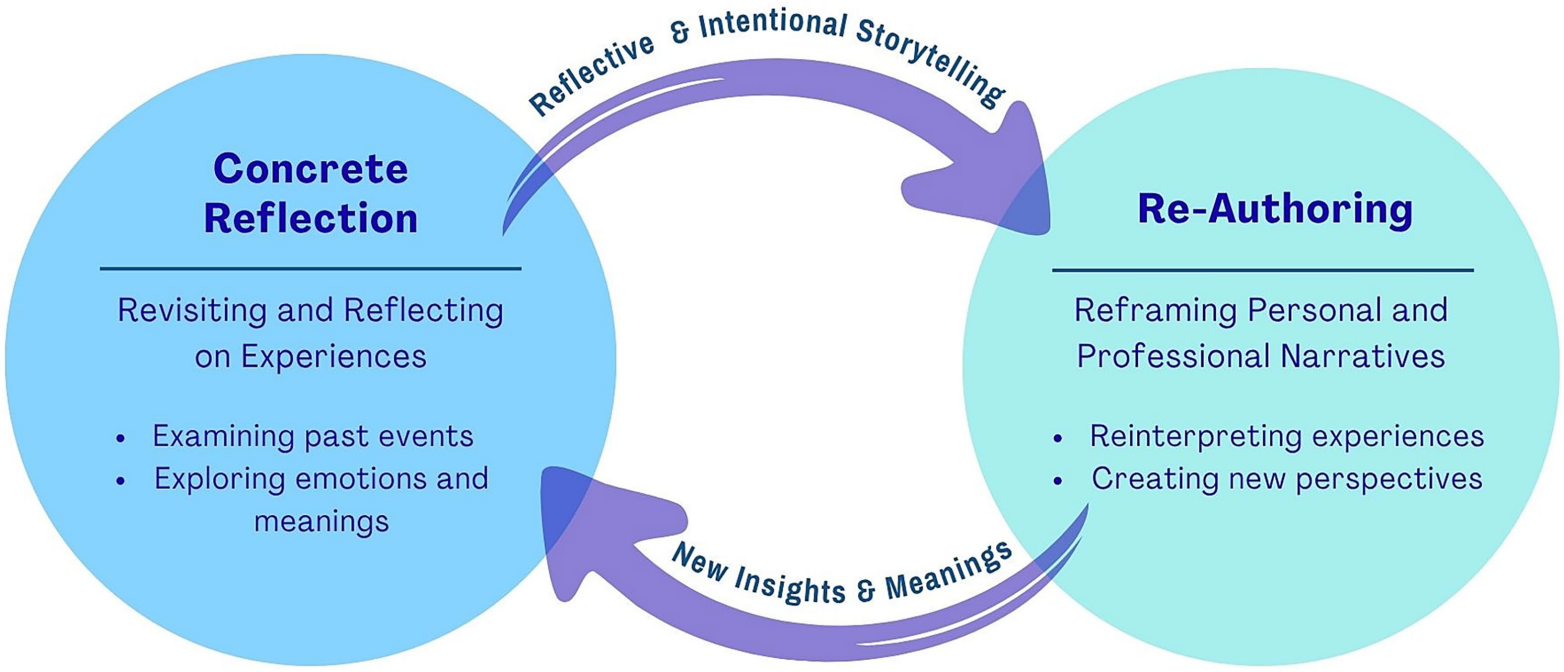
Conceptual relationship between concrete reflection and re-authoring in storytelling, showing how revisiting past events through storytelling supports the reinterpretation and reframing of personal and professional narratives of lived experiences.

Phenomenology also recognizes that experience is subjective and shaped by context, including one’s professional journey, sense of community, and prior challenges in practice (30). Through first-person accounts, this approach supports an in-depth understanding of how participants experience preparing their stories, sharing them with readers, and reflecting afterward. Importantly, phenomenology aligns with the concepts of concrete reflection and re-authoring in narrative research. Concrete reflection occurs when individuals revisit and articulate lived experiences through telling and retelling their stories, allowing deeper interpretation of their meaning (31–33). This reflective retelling can support re-authoring, where individuals reinterpret and reshape their personal and professional narrative over time (24). The interconnected mechanisms of concrete reflection and re-authoring (Figure 1) can transform an individual’s story from a negative narrative into an empowered and preferred account. In the context of a Living Library, storytelling may prompt rural healthcare professionals to re-examine past experiences, affirm their values, and develop renewed meaning in their work. Thus, a phenomenological lens is well suited to capture how being a human book involves reflection, meaning-making, and shifts in professional understanding, while recognizing participants as experts of their own lived and professional experiences. This framework focuses on the transformative aspects of storytelling, capturing subtle shifts in perspectives, meaning-making, and connection that are central to the “human book” experience.

### 2.2 Study design and context

This exploratory qualitative study was conducted within a rural medical education program that incorporated four Living Library events. A phenomenological approach informed the design and analysis of this study, focusing on how rural healthcare professionals experienced telling their personal stories as human books in a Living Library. *The Library of Life: Stories of Rural Medicine* events were held annually since 2021. Across four Living Library events, twenty-nine (29) rural healthcare professionals and community members participated as “human books,” sharing self-determined snippets of their lived experiences of rural life and practice with medical learners (“readers”), in facilitated conversations and respectful, active listening. The events followed a 3-rotations format, each rotation lasted 30 minutes, with 20 minutes for sharing of the books’ narratives, 10 minutes for an interactive question-and-answer portion, and 5 minutes for transition.

### 2.3 Participants and data collection

Human books who shared at a Living Library event were invited via email to be interviewed. Participants included rural generalist physicians, physician trainees, allied healthcare providers (nurses and physician assistants), Indigenous elders, and a spouse of a rural physician. Participation was voluntary, and informed consent was obtained. This research project was approved by the Conjoint Health Research Ethics Board (CHREB) of the University of Calgary.

Data were collected through semi-structured, one-to-one interviews conducted by two experienced interviewers (PF, EM). The interview guide with the semi-structured questions, is available in the Appendix. To ensure a conversational tone, the interview guide was applied flexibly and was informed by sensitive research considerations (34). Interviews explored participants’ motivations for sharing their stories, emotional preparation, experiences of storytelling, interactions with readers, and perceived personal and professional impacts. The interviews were conducted via Zoom and lasted between 45 and 60 minutes. They were audio-recorded and transcribed verbatim by a professional transcription service, which removed any identifiers.

### 2.4 Data analysis

Thematic analysis was completed by two researchers (GP, KT), and followed the six steps outlined by Braun and Clarke (35, 36): (1) reading transcripts repeatedly to develop familiarity with the data; (2) generating initial codes to capture meaningful concepts; (3) gathering codes into clusters of shared meaning to form themes; (4) refining themes together to create a standard coding scheme; (5) recoding transcripts using the set coding scheme; and (6) selecting quotes reflective of the themes. Three iterations of coding were performed; first, a small proportion (24%) of transcripts were used to construct the initial codebook (GP, KT); second, themes were checked and refined together with supervising author (AJ) to develop the final coding scheme; third, all transcripts were recoded using the set coding scheme. The two-person coding approach helped to reduce individual bias, enhanced reliability, and ensured richer evaluation of the transcripts.

### 2.5 Reflexivity and positionality

The study team comprised of medical educators who designed and implemented the Living Library, a learner (KT) who helped with the data analysis, and two interviewers (PF, EM). AJ is the associate dean for distributed learning and rural initiatives of the medical school. KT is an undergraduate student raised in a rural community, with an interest in rural medicine. GP supports the various scholarship activities of the distributed teaching faculty, preceptors, and medical learners. Reflexive thematic analysis was used as the primary analytical approach where researchers critically reflect on their values and assumptions throughout the research process (37). The research team may have held bias based on their own assumptions and experiences of the Living Library events. Reflexivity was maintained through ongoing team discussions throughout the study (38), which supported critical reflection on interpretations and enhanced analytic depth.

## 3 Results

Among the 29 “human books,” there were 20 (69%) physicians, 3 (10%) medical residents, 1 (3%) nurse, 1 (3%) physician assistant and 3 (10%) members of the community. Among the physicians, 16 (55%) had rural generalist practice, 2 (7%) were specialists with rural practice, 1 (3%) was locum physician and 1 (3%) had a hybrid rural–urban practice. Most physicians (45%, *n* = 13) were in the mid-career stage, while 4 (14%) and 9 (31%) were in early career and late career stages, respectively; 3 (10%) of physicians also held academic leadership roles. Among the community members were an Indigenous Elder and a spouse of a rural physician. Six (21%) participants were persons of color and 3 (10%) were Indigenous. There were slightly more females (59%, *n* = 17) than males (41%, *n* = 12). Nineteen (66%) consented to be interviewed and all provided rich insight into their experiences. Phenomenological approach emphasizes depth over breadth, with 9–17 interviews required to reach thematic sufficiency (39).

### 3.1 Thematic analysis

Analysis revealed three interrelated themes that describe the meaning of being a human book: (i) participants felt a responsibility to share their experiences to inform and inspire future rural practitioners; (ii) story preparation was emotionally challenging and required psychological readiness; and (iii) storytelling offered personal benefits, including connection, reframing past experiences, strengthening personal networks, and renewed sense of purpose and meaning in their work, which enhanced professional identity. Together, these themes illustrate that storytelling was experienced not only as an educational activity but also as a reflective and meaningful professional experience.

#### 3.1.1 Theme 1: human books felt a responsibility to share their stories

In crafting and preparing their stories, participants described a strong sense of responsibility to share their insights and experiences with medical learners, representing rural practice honestly to inspire future rural practitioners. Participants saw themselves as advocates for rural practice and felt accountable for conveying honest, meaningful stories that could influence learners’ perspectives. Many viewed their role as an opportunity to represent rural practice authentically and to challenge misconceptions about working in rural settings. They felt that their lived experiences could inspire learners and promote interest in rural healthcare. This sense of responsibility was closely linked to professional identity. Participants described storytelling as an extension of their role as educators and mentors, reinforcing their commitment to rural healthcare and future workforce development.

(i) The participants felt it was their responsibility to inspire future rural physicians and health practitioners.

*“… I work exclusively in rural sites in Alberta. I graduated from medical school in 2005, but my practice nowadays is split mainly between emergency medicine and GP anesthesia which also encompasses rural critical care… I’m passionate about teaching and I feel like the opportunities to have a physician career outside of cities is not well covered by most medical schools in Canada. And certainly, recruitment to rural communities needs all the help it can get. And I think speaking to students early on in their training and planting that seed will open their eyes to possibilities that they may not otherwise consider until they’re too far into their training.”* (Participant 14)

*“I had a chance to tell my story and to talk to future physicians and hopefully, they saw value in it and that they will be inspired.”* (Participant 5)

(ii) The participants recognized the need for more rural health practitioners and hoped their stories would resonate with the readers and inspire medical learners to take a similar path.

*“I did it thinking about medical students and trying to convince them that they wanted to come do an elective and then hopefully join rural medicine. So, I picked things that would show them that it's not a job as much as it's a fun way to spend your life. I picked my stories based on what I thought would challenge them to challenge themselves.”* (Participant 4)

*“I tried to be representative of my journey, my pathway, how it is that I got to be where I was and the things I was doing and how it is that from my perspective I was able to integrate doing the variety of things that I did, which I think is somewhat appealing. And particularly in the context of somebody who works both within academic medicine and works in a rural community.”* (Participant 11)

(iii) The participants felt responsible to pass on knowledge to the next generation of rural doctors. Most of the human books felt it was part of their responsibility as a rural health professional to share their experiences and expertise with future rural health care professionals. They felt fulfilled through this sharing of knowledge.

*“… trying to pass on knowledge and experience to the next generation. And I've always sort of felt that was a responsibility and one aspect of extended health care that I'm good at that is being able to connect with learners and educate them and whatnot. And so it kind of ticks that box for me. I feel good when I'm able to help people grow.”* (Participant 7)

*“There’s a whole bunch of people in my category – semi-retired or retired or should-be retired. And of course, we need to be replaced by newer and younger models. And unless what we do is inculcate into those newer and younger models the passion in compassion and the enthusiasm for the work that we do, unless we impart that to them, then we’re leaving it up to chance. So that’s not what I’m going to do. I know I’m going to be replaced, and I have been replaced in the capacity of physician in a rural community. But I don’t want to be replaced by substandard passion.”* (Participant 18)

#### 3.1.2 Theme 2: story preparation was emotionally challenging and required psychological readiness

Participants described preparing to be a human book as a challenging process. They reported revisiting personal and professional experiences, some of which were emotionally charged, involved challenges, sacrifices, and moments of growth. Storytelling was described as an intentional act requiring psychological preparation and readiness rather than a spontaneous recounting of events, before engaging with readers. They found preparing a story to be a process of reflecting on their lifepath which was emotional at times due to retelling of sensitive experiences. The participants used a variety of psychological preparation techniques to craft their stories.

(i) Some participants found preparing and telling their stories to be an emotional experience. Human books recalled using techniques to manage their emotions including rehearsing the story beforehand, as depicted in the quote, as well as usage of spirituality and grounding techniques.

*“So the part that was emotional, I rehearsed it a little bit. Actually, while I was driving in for Library of Life. Yes, I kind of, I rehearsed it to myself, told it a couple of times and I think that took some of the raw edge of the emotion off before telling the story with the listeners.”* (Participant 8)

*“When you’re telling a story a lot of emotions start to come out that you did not anticipate. And some of the students were asking questions about racism and lack of diversity in small towns here. And of course, I have my personal experiences with that so I was able to answer those questions, but it did trigger some memories. Some were good memories; some were not so good. So, yeah. It definitely brought up some emotions.”* (Participant 15)

(ii) Human books reflected on what to include, how to present their experiences, and how much vulnerability to offer, in their stories.

*“When people start asking you questions about your practice style or where you are in your life and then how your life has changed over the last, you know, number of years. For me it has changed a lot. So I had a very long story to tell but I told it to them in small pieces. And then so that it can be – they can relate to it, basically. So I did divide it up into my early career and then mid-career and then my current part of what I’m doing.”* (Participant 15)

*“So it was good for me to sort of like reflect on the most important parts of my story that I wanted to share and to sort of identify some of the emotions that came along with it so that I would be kind of prepared if I did feel emotional during the presentation as well. So sort of like identifying in advance what I wanted to say and then which parts might be hard to say and thinking about why and practicing them a bit. And then just sort of accepting that, I guess—that if that happens that’s OK.”* (Participant 19)

(iii) The participants reflected on their experiences while crafting a story. They noted that crafting a story required looking at their lifepath and career as one cohesive narrative. Human books then reflected on their experiences in a more focused manner.

*“I found it interesting just preparing my story and just thinking it through and thinking about what I wanted to say. And even after I gave the presentation, you know, it just brought up some reflections about, you know, what is important to me in terms of my practice and what do I value.”* (Participant 9)

*“…kind of a higher viewpoint reflection on ways I've contributed, things I've learned about myself, about society. Yes, you always think about that and I do jot notes down from time-to-time on interesting cases or reflections. But this was kind of a unique opportunity to frame some of my experiences with a specific direction or a specific kind of theme.”* (Participant 8)

#### 3.1.3 Theme 3: storytelling was a transformative experience, supporting deep reflection and meaning-making and renewed purpose

Participants described multiple personal benefits from being a human book. A key outcome was the emotional connection formed with readers, which created a sense of being heard, valued, and understood. These interactions were described as meaningful and affirming. Participants also reported reframing past challenges through storytelling. Experiences that were once viewed as difficult were reconsidered as meaningful parts of their professional journey. Several participants noted that retelling their stories across multiple sessions deepened reflection over time. The process of repeated storytelling encouraged them to organize their thoughts, clarify meanings, and become more aware of how their experiences had shaped their professional paths. Repeated engagement in storytelling also allowed participants to refine their narrative skills and communicate their experiences more clearly. Participants felt that storytelling fostered validation their work and helped strengthen their own professional networks.

(i) Storytelling was experienced as a relational exchange rather than a one-way teaching activity, and the human books valued the opportunity to connect with the learners. They described experiencing deeper connections and sharing in the Living Library format than during regular style conferences or everyday life. Telling their stories in a non-judgmental space allowed participants to feel heard and validated. The attentive listening of readers created a sense of safety that enabled openness and authenticity. This connection with students provided value to the books experience.

*“I think when somebody reads your book, they do remember your story and it's quite a—you share some sort of deeper details of your life that you don't normally just share in a brief conversation otherwise, meeting somebody at say a conference or another social setting, we don't normally get into these sort of in-depth details of our lives.”* (Participant 1)

*“I felt very, very encouraged by the folks that came around to my table. I listened very carefully to what a lot of them had to say. And a lot of them didn’t really indicate their misgivings about proceeding into following in a rural practice or into medicine as such. A lot of them were also premed students and enthusiastically wished that they were already in medicine. And so these were highly motivated young people who I think with the right training and mentorship – mentorship is important—they would actually be the leaders of tomorrow. I don’t feel that our profession is going to be impoverished by any of the inputs that I heard. There was no negativity. There were no people who decried the challenges faced by all of us in this province given the political situation and the tough working conditions in rural Alberta as an example”* (Participant 18)

(ii) The experience allowed the participants to assign increased meaning to their stories through reflection. This process of re-storying supported meaning-making, helping participants integrate challenges and reaffirm the value of their rural practice.

*“But having to kind of put together in my own head this kind of – I think I called it the long and winding road. Because it was a long and winding road. I mean, I didn’t go into medicine till I was in my late 20s. And so even my life before medicine has a whole lot to do with who I am as a physician and yet had nothing to do—well, had everything to do with medicine but people in medicine wouldn’t have thought that that was the case.”* (Participant 13)

*“It did bring me joy and self-confidence… through more on the self-reflection, there was definitely just identification of how I do take on challenges. And, you know, I think of that now often when I’m taking on new roles and new challenges. I think that was definitely a turning point.”* (Participant 10)

(iii) Through telling and retelling their stories, human books described re-examining past experiences and gaining new perspectives and meaning, with a renewed sense of purpose.

*“I had a chance to think and clarify some of my values and priorities of my work. It was nice to be able to share my insights and knowledge with the students. I had brought to the table some of the books that I found particularly kind of useful and inspiring in my work. … I don’t recall exactly everything that I said. But I do know that I was talking about working with indigenous populations and why that’s important to me and just also talking a little bit about my career in rural medicine and, like, why I chose rural medicine.”* (Participant 9)

*“Looking back on part of my career and how that had worked out so far… I think it was all very, very positive. And the fact that I then got to do that a couple of times and then reflect upon it, again on the way home after I had been asked a bunch of questions and sort of putting those things together.”* (Participant 11)

(iv) One participant conveyed their passion for serving the rural community and reinforced their professional identity as a rural practitioner.

*“There are many things to be passionate about [rural medicine]. And my feeling is that if in fact your contribution to the wellbeing of others involves serving in the capacity of a healthcare provider, then be passionate about it. A lot of people talk about how poorly we’re paid or how exorbitantly we are paid or the poor conditions in which we have to work or the difficulties that we face when we work 60 to 80-hour weeks, which is something that I did during my time as a rural practitioner. … But that’s not what it’s all about. What it’s all about is serving people to the best of your ability with passion and with compassion. So that was the topic that I chose. What’s there to be compassionate about? And medicine is about passionately pursuing your desire to work with people, to help people, to live your calling within the privilege endowed upon us by society, who are called doctor.”* (Participant 18)

(v) Participants described moments of emotional connection with readers and a sense of shared understanding across differences in experience and career stage. These interactions generated feelings of satisfaction, pride, and renewed purpose. Sharing their stories reinforced participants’ professional identity and provided personal fulfilment through contributing to learners’ interest in rural healthcare.

*“I was able to rethink of some of my past experiences on other types of roles I've had and how they're similar to what we want to achieve as a health provider. So yes, I guess being able to rethink some of these experiences and make it meaningful, I think was what I gained.”* (Participant 6)

*“I think it felt empowering, kind of, to sort of have a platform to share some of the difficulties that I faced. But also kind of the way that I’ve been able to move forward. So it was good for me both in the reflection and in the actual experience of sharing it and especially of sharing it again. And I think as I have given many presentations about different things that I have done, that one was helpful for me.”* (Participant 19)

(vi) The participants noted that the event offered not just an opportunity to hone their storytelling skills, but they appreciated how valuable storytelling can be. They mentioned that their story touched not only medical learners, but also other people not present at the event, including professional colleagues, who have reached out about the story long after the event concluded. This provided further validation of their work and helped strengthen their own professional networks.

*“I have to confess that I've been learning more about storytelling and how impactful stories can be and working to be a better storyteller. So, it was an opportunity to practice.”* (Participant 5)

*“I thought it was effective for those who were present. And it was interesting as a book too to sort of prepare the story. It was also interesting because I did it twice.* … *And so, I think that it heightened our relationships too within the hospital… And I also have had people, like even physicians, say that they heard [what I’ve talked about] or whatever. And then they messaged me to talk about how to get involved in medical assistance in dying. So, I feel like, yeah, it has different sorts of impacts that I hadn’t really thought about beyond just that initial audience.” (Participant 19)*

## 4 Discussion

This qualitative study explored what it means for rural healthcare professionals to serve as human books in a Living Library within rural medical education. The Living Library setting presents unique conditions that may shape the storyteller experience. Human books are invited to share their stories repeatedly, often with multiple readers, and to respond to questions in real time. This may create opportunities for affirmation, connection, and validation, particularly when stories are received without judgement. At the same time, it may involve emotional labor, vulnerability, and the need for psychological preparation. From 19 transcripts, the study uncovered three related themes, (i) responsibility to share and inspire, (ii) emotionally challenging preparation, and (iii) personal benefits through connection, reflection and reframing.

The first theme reported on the strong sense of responsibility to share and inspire felt by the human books and pointed to how rural healthcare professionals see themselves as role models and advocates for rural practice. In the context of Canadian rural medical education, where recruitment and retention of the rural workforce remain ongoing challenges (40, 41), this finding is significant. Human books did not simply provide information about rural careers. They offered authentic, experience-based insights that helped medical learners across pre-medical, undergraduate, and postgraduate stages better understand the realities and rewards of rural healthcare. This aligns with the broader literature on narratives in medical education (5–7), which suggests that lived stories can challenge misconceptions and foster more informed career perspectives (4, 19). From a medical education standpoint, integrating rural clinicians as storytellers to complement rural curricula may strengthen early exposure to rural role models and support interest in future rural practice.

The second theme described the emotional burden felt by human books in crafting their stories. Preparing their personal stories prompted participants to revisit key moments in their professional journeys, including challenges, values, and motivations for working in rural settings. Revisiting emotionally significant experiences made participants vulnerable and required psychological readiness in order to present a coherent sense of self to others. This implies thoughtful story development, and storytelling becomes an intentional, structured space for emotional processing rather than a simple recounting of events (33). Hence, storytelling can be a tool for transformation and influence sense of identity. In rural and marginalized communities in Canada, where clinicians often work in complex and resource-limited contexts, such reflective practice opportunities are rarely formalized within professional development.

The last theme focused on the personal benefits reported by participants, including building connections, reframing of past experiences, and refining storytelling skills (20), which expand current understanding of narrative-based education. They described storytelling as an opportunity to examine past events, foster connection with listeners and other colleagues, and reinforce their own identities as members of a rural healthcare community of practice. The act of being heard by engaged medical learners also created a sense of recognition and connection, which may be particularly meaningful in rural practice settings where professional isolation is common. Moreover, the sharing and retelling of stories multiple times enabled concrete reflection (32, 33). Concrete and focused reflection serves as a mechanism for re-authoring, which allows participants to revisit past events, ponder on their significance, and make new meaning of their experiences (24, 25). Re-authoring enables participants to build an alternative storyline that is more accurate to their strengths and values and shift the narrative via meaning-making. In rural settings where professional isolation, emotional strain, and high workloads are common, initiatives that allow for intentional and introspective storytelling can foster agency and resilience, improve wellbeing, and boost professional satisfaction. Storytelling also appears to strengthen professional networks and mentorship by cultivating a sense of belonging and shared identity.

This study contributes to the literature by shifting the focus from learner outcomes to the impact of storytelling for the storyteller themselves. Most existing literature on Living Libraries evaluates impacts on learners, such as increased empathy and attitude change (15–17, 19). This research demonstrates that being a “human book” can also be a meaningful experience for the storytellers involved, validating the value of stories not only for those who listen but also for those who share them. This study also fulfills one of the recommendations made by Dobreski and Huang (20) for more research into different types of human books and confirms that storytelling is a transformative and empowering journey that acts as a catalyst for professional renewal, rekindling purpose and providing validation among rural healthcare professionals.

These findings have important implications for rural medical education in Canada. Narrative-based learning can help bridge the gap between formal training and realities of rural medicine. Living Libraries can be strategically embedded within rural curriculum to inspire rural healthcare interest, encourage social accountability and support workforce pipeline development (19). By exposing medical learners at all stages of training (pre-medical, undergraduate and postgraduate) to authentic rural narratives, educators can promote a more realistic understanding of rural practice, where learners gain insights into the seldom noticed but noteworthy facets of rural practice (e.g., the quiet bravery rural physicians demonstrate every day in their practice or their advocacy and resilience amid resource-constrained settings). Living Libraries can also provide the reflective space for rural practitioners to reinterpret their experiences over time and construct renewed meaning in their work. However, institutions should recognize the emotional labor involved in storytelling, such as revisiting challenges of rural practice, recounting personal struggles including racism, family separation and grief, as well as managing performance anxiety. Being mindful of the emotional strain associated with storytelling, organizers can ensure that human books are supported when sharing more vulnerable aspects of their journeys and identities by providing appropriate preparation, psychological assistance, and debriefing.

The implications also extend to similar underserved regions outside Canada. Many countries face similar challenges in recruiting and retaining healthcare professionals in rural areas (42–45). The Living Library is applicable to many environments as the resources to run these events are not costly. Indeed, the primary resources required are the stories of the storytellers, and their willingness to share these with the learners (16, 19). Integrating structured storytelling initiatives can support values-based learning, empathy development, and early interest in rural careers, which are critical for strengthening the future rural healthcare workforce. This research provides evidence on the potential contribution of “human books” for recruiting doctors to work in rural communities. By positioning rural clinicians as experts by experience, this approach supports community-informed education and aligns with global calls for socially responsive health professions training (46–48).

### 4.1 Strengths and limitations

This study has several strengths. First, the use of the phenomenological approach allowed for an in-depth understanding of how rural healthcare professionals experience being “human books,” focusing on meaning rather than surface description. Second, the participants represented different phases of rural practice and practice environments, which enriched the range of perspectives on storytelling within rural medical education. Third, the study is based in a real educational context where medical learners at different stages of training engage with practicing professionals, increasing the relevance of the findings for providing rural medical education opportunities in Canada and comparable regions globally. However, the sample size was relatively small and specific to rural practice context, which may limit transferability to other educational or cultural settings. Participation in the Living Library was voluntary; the human books may have been more comfortable with reflection and storytelling than other rural healthcare professionals, introducing potential selection bias. In addition, data relied on self-reported experiences, which may be influenced by recall and social desirability. For future studies, the recall bias can be mitigated by conducting the data collection within a timeframe closer to the Living Library event completion. The social desirability bias may be inherent in the work (49), but it may be mitigated by using a trained facilitator to conduct the interviews or focus groups, phrasing questions neutrally to avoid leading answers and emotionally charged language, or using indirect questioning techniques. Social desirability bias within the Living Library may itself be a target of future research.

## 5 Conclusion

The Living Library model offers an innovative educational approach that supports both learners and clinicians. It emphasizes storytelling not only as a teaching modality, but also as a valuable form of reflexive practice for those who share their stories. For rural healthcare professionals, being a “human book” represents an impactful educational role rooted in a strong sense of responsibility, self-reflection, and personal growth. Sharing their stories was not only an act of teaching but also a process that empowers the storytellers through the validation of their unique career paths and the opportunity to advocate for their communities’ needs. For medical educators, recognizing the dual benefits for learners and storytellers can inform the design of narrative-based programs that strengthen rural medical education, while valuing the voices and experiences of rural healthcare professionals. Living Libraries can complement traditional curriculum and support workforce development in rural and marginalized contexts worldwide.

## Data Availability

The datasets presented in this article are not readily available because the authors could not make the data available to the readers due to the sensitive nature of the data. The interactions and other situational experiences mentioned by the participants in the transcripts could make the individual personalities involved easily identifiable, despite applying great amounts of anonymization. Requests to access the datasets should be directed to grace.perez@ucalgary.ca.

## Appendix A

### Interview guide

1. What made you decide to participate in the Library of Life event as a Book?
2. Had you previously thought of your experiences as a story? Why did you choose the story that you shared?
3. What was it like putting your experiences into a story type of format? How did you decide what to put in or keep out?
4. Did this experience change anything about how you view your own story? What insights into your own story did you gain after discussing it?
5. Did the readers have any questions or reflections on your story that surprised you?
6. Did you have any emotional or psychological struggles while telling the story? How did you overcome this feeling as you were sharing?
7. How did you benefit from your participation? What did you gain from it personally? (e.g., joy/satisfaction from self-reflection?)
8. Were there any questions that we did not ask you, that you would have liked us to ask?
